# Long noncoding RNA LINC00314 facilitates osteogenic differentiation of adipose-derived stem cells through the hsa-miR-129-5p/GRM5 axis via the Wnt signaling pathway

**DOI:** 10.1186/s13287-020-01754-z

**Published:** 2020-06-17

**Authors:** Zheng-liang Shi, Hua Zhang, Zhi-yong Fan, Wei Ma, Yong-zhou Song, Ming Li, Tong-qiu Li, Shu-xing Cao, Guo-jun Feng

**Affiliations:** Department of Orthopedics, The Second Hospital of Hebei Medical University, No. 215, Hepingxi Road, Shijiazhuang, 050000 Hebei Province China

**Keywords:** LINC00314, miR-129-5p, GRM5, ADSCs, Osteogenic differentiation

## Abstract

**Background:**

Many studies have shown that long noncoding RNAs (lncRNAs) are closely related to the stimulation of osteogenic differentiation of adipose-derived stem cells (ADSCs) and the prevention of osteoporosis. Current research aimed to investigate the novel lncRNA and explored the function and molecular mechanism of the LINC00314/miR-129-5p/GRM5 axis in regulating osteogenic differentiation of ADSCs.

**Methods:**

LncRNA and miRNA sequencing was performed in normal and osteogenic differentiation-induced ADSCs (osteogenic group). Abnormally expressed lncRNAs and miRNAs were obtained by the R software and the relative expression of LINC00314, miR-129-5p, and GRM5 during osteogenic induction was measured by RT-PCR. ADSCs were then transfected with pcDNA3.1-sh-LINC00314 and agomiR-129-5p. Alizarin red staining (ARS) and alkaline phosphatase (ALP) staining were performed to identify the mechanism of the LINC00314/miR-129-5p/GRM5 axis in regulating osteogenic differentiation of ADSCs.

**Results:**

LINC00314 was significantly upregulated in the group of osteogenic-induced ADSCs. LINC00314 and GRM5 mimics increased the early and late markers of osteogenic differentiation, which manifest in not only the markedly increased ALP activity but also higher calcium deposition, while miR-129-5p mimic had the opposite effects. LINC00314 directly targeted miR-129-5p through luciferase reporter assay, and miR-129-5p suppressed GRM5 expression. Moreover, the LINC00314/miR-129-5p/GRM5 regulatory axis activated the Wnt/β-catenin signaling pathway.

**Conclusions:**

LINC00314 confers contributory function in the osteogenic differentiation of ADSCs and thus the LINC00314/miR-129-5p/GRM5 axis may be a novel mechanism for osteogenic-related disease.

## Background

Osteoporosis is a serious degenerative disease characterized by the reduction of bone mass and destruction of the bone microstructure [1, 2]. Osteoporosis can result in fractures and may increase economic cost and societal burden [3]. Previously, bone marrow mesenchymal stem cells (BMSCs) were administered as seed cells to improve BMD and used for tissue engineering [4]. However, the proliferation ability of BMSCs was limited, especially in older patients. Adipose-derived stem cells (ADSCs) could be a potential alternative for BMSCs due to their advantages of being easy to obtain and widely sourced [5, 6].

Long noncoding RNAs (lncRNAs) belongs to a family of small RNAs, which are often 200 or more nucleotides long and play crucial roles in bone disease [7]. Zhang et al. [8] reported that lncRNA NKILA indirectly regulates RXFP1/AKT signaling pathway to regulate osteogenesis. Another study also found lncRNA HOTAIRM1 directly targeting with JNK/AP-1 signaling pathway to control the osteogenic differentiation [9]. Wu et al. [10] analyzed the differentially expressed long noncoding RNAs in the normal and induced groups and found that HIF1A-AS2 has a positive role in promoting ADSCs osteogenic differentiation through the PI3K/Akt signaling pathway. LncRNA LINC00314 is located on chromosome 21 with open reading frame 94. Currently, no study has explored the role and mechanism of LINC00314 in the regulation of ADSC osteogenic differentiation.

There is now overwhelming evidence that alterations in microRNA (miRNA) expression levels are linked to osteogenic differentiation [11]. Several miRNAs have been identified to have potential roles in promoting or inhibiting the osteogenic differentiation of ADSCs [12]. Li et al. [12] found that miR-154-5p could directly bind to the 3′-UTR of Wnt11 and thus inhibit the osteogenic differentiation. Liu et al. [11] revealed that miR-145-5p suppresses osteogenic differentiation of ADSCs by targeting semaphorin 3A.

MiR-129-5p participates in regulating the proliferation, migration, and invasion of retinoblastoma cells and affects spinal cord injury [13, 14]. In a study on the regulation of osteogenic differentiation, Valenti et al. [15] found that physical exercise modulates miR-129-5p expression in progenitor cells and thus promotes osteogenesis. Further study also revealed that miR-129-5p could regulate osteoblast differentiation of BMSCs.

Metabotropic glutamate receptor 5, named GRM5, has been shown to activate phospholipase C and participate in multiple biological processes [16]. Many studies have shown the Wnt/β-catenin signaling pathway participant into the osteogenic differentiation of ADSCs. However, whether LINC00314 could activate the Wnt/β-catenin signaling pathway and stimulate osteogenic differentiation of ADSCs remains unclear.

In this study, LINC00314 was found to be abnormally expressed during the osteogenic differentiation of ADSCs through gene chip and further confirmed by qRT-PCR. Further bioinformatics analysis revealed LINC00314 directly regulating with miR-129-5p and its targeting gene GRM5.

## Methods

### Tissue specimens and ADSC cultures

Human adipose tissue was collected from patients who prepared for total knee arthroplasty. Each patient signed an informed consent form, and this research was approved by the ethics committee of the Second Hospital of Hebei Medical University. Collected adipose tissue was washed with PBS three times and cut into sections. Then, these sections were digested with 0.1% type I collagenase (Gibco, Grand Island, NY, USA) at 37 °C for 1 h. Next, we added 5 mL DMEM containing 10% fetal bovine serum (FBS, Thermo Fisher Scientific, Inc., USA) to inhibit the type I collagenase activity. The cell suspension was centrifuged at 300×*g* to collect the cells. Cells were cultured in high-glucose Dulbecco’s modified Eagle medium (DMEM, Gibco, Grand Island, NY, USA) with 20% FBS and 1% penicillin at 5% CO_2_ and 37 °C. ADSCs at passage 3 were used for subsequent studies. A previous study revealed that less than 5% of the ADSCs showed senescence when expanded to generation 10 [17].

### ADSCs identification

To identify ADSC surface markers, ADSCs at passage 3 were collected in tubes at 4 × 10^5^/tube. Next, anti-CD31-PE, anti-CD45-PE, anti-CD44-FITC, anti-CD29-FITC, anti-CD73-PE, anti-CD90-FITC, and anti-CD105-PE (all from BD Biosciences, San Jose, CA, USA) antibodies were incubated. Then, ADSCs in solution were identified by a FACS Calibur flow cytometer (Becton-Dickinson, Franklin Lakes, NJ, USA).

Trilineage differentiation was performed to show the differentiation potency of ADSCs. In brief, ADSCs were seeded into 6-well plates (5.0 × 10^5^ cells/well) with normal medium until cell confluence reached approximately 70%. The nonadherent cells were removed by replacing the medium, and the attached cells were cultured until confluence. The cells were then grown for 21 days in the adipogenic, osteogenic, and chondrogenic medium (Cyagen, Guangzhou, China). Alizarin red S staining (ARS, Solarbio, Beijing, China) was performed to assess osteogenic differentiation. Adipogenic differentiation was visualized using Oil Red O (Sigma-Aldrich, St. Louis, MO, USA) staining. Alcian Blue (Sigma-Aldrich, St. Louis, MO, USA) staining was used to assess chondrogenic differentiation.

### Microarray

Microarray analyses of lncRNA and microRNA expression were performed as described previously [18]. Briefly, total RNA from the normal and induced groups was extracted by TRIzol as described previously. cDNA was synthesized, labeled with fluorescent dye, and hybridized with a lncRNA Human Gene Expression Microarray v4.0 (4 × 180 K; Cloud-Seq Biotech, Shanghai, China) platform (LC Sciences, Houston, TX, USA) for lncRNA and microRNA, respectively. Differentially expressed lncRNAs and mRNAs were obtained by the limma package. Moreover, heatmaps and volcano plots were generated by using Bioconductor (http://www.bioiconductor.org) in R software (Free Software Foundation Inc., Boston, MA, USA).

### Bioinformatic analysis

First, the limma package was utilized to identify differentially expressed lncRNAs and miRNAs in the induced and non-induced groups using the screening criteria |logFC (foldchange)| ≥ 1 and adjusted *P* < 0.05. Cluster enrichment of gene ontology (GO) and Kyoto Encyclopedia of Genes and Genomes (KEGG) pathway was performed using the Database for Annotation, Visualization and Integrated Discovery (DAVID) database (https://david.ncifcrf.gov/).

The GO terms including three categories: biological process (BP), cellular component (CC), and molecular function (MF). Target genes network were constructed by the Search Tool for the Retrieval of Interacting Genes (STRING) database.

The interaction of miRNAs with LINC00314 was predicted from a reliable online miRNA reference database, miRcode (http://www.mircode.org/). The prediction of target mRNAs of miRNAs was performed using three databases, miRDB (http://www.mirdb.org/), miRTarBase (http://mirtarbase.mbc.nctu.edu.tw), and TargetScan (http://www.targetscan.org) and the miRNA profile. The ceRNA network was visualized by Cytoscape (https://cytoscape.org/).

### Cell transfection

LINC00314, sh-LINC00314, agomiR-129-5p, pcDNA3.1-LINC00314, antagomir-129-5p, pcDNA3.1-GRM5, and their respective negative control (NC) came from GenePharma (Shanghai, China). In brief, lipofectamine 3000 (Invitrogen, Carlsbad, CA, USA) and transfection object were used to transfect for 10 min. The transfection rate of each reagent was confirmed to be ≥ 90%. To inhibit the effects of WNT activation, XAV939 (MCE, Shanghai, China) was utilized to pre-treat ADSCs before pcDNA3.1-GRM5 treatment. XAV939 was used to treat ADSCs at a final concentration of 10 μM according to a previous study [19].

### qRT-PCR

Trizol reagent (Takara Bio Inc., Otsu, Shiga, Japan) was applied for total RNA extraction from cells. Afterwards, mRNA levels were determined using the SYBR Green qPCR Mix Kit on the ABI 7500 Fast Real-Time PCR System (Thermo Fisher Scientific Inc., Waltham, MA, USA). All primers were purchased from Invitrogen (Shanghai, China). Glyceraldehyde-3-phosphate dehydrogenase (GAPDH) and U6 were utilized for internal control as appropriate. Sequences of primers are presented in Table 1. The relative quantitative expression of interest genes was expressed as fold change (2− ΔΔCt method). ∆∆Ct = [Ct (target gene) − Ct (GAPDH/U6)] experimental group − [Ct (target gene) − Ct (GAPDH/U6)] control group. Each sample was examined in triplicate.

**Table 1:** Primer sequences for qRT-PCR

Gene	Forward primer 5′-3′	Reverse primer 5′-3′
GAPDH	GACAGTCAGCCGCATCTTCT	GCGCCCAATACGACCAAATC
MiR-129-5p	GGCTTTTTGCGGTCTGG	CAGTGCGTGTCGTGGAGT
RUNX2	CCTTCAAGGTGGTAGCCCTC	CCCTAAATCACTGAGGCGGT
Osterix	AGACCTCCAGAGAGGAGAGAC	GGGGACTGGAGCCATAGTGA
Osteocalcin	AATAGCCCTGGCAGATTCCC	CTCTCATGGTGTCTCGGTGG
LINC00314	GATCTATTGTTTAGCCAT	ATGGCUAAACAAUAGAUC
GRM5	AGATCTGTGGCTCAGTTCCTTT	AATCACTATGAATCCCTGCACCT

### Western blot analysis

The expression level of osteogenic differentiation-related genes was determined by Western blot analysis. The equivalent cracking solution was added to phenylmethyl sulfonylfluoride until the concentration was 1 mM (No. ST506, Beyotime Biotechnology Co., Ltd., Shanghai, China). The transfected cells were lysed with Western and IP cell lysates. After full cracking, the cells were centrifuged at 10000–14000×*g* for 3–5 min with the supernatant collected. Bicinchoninic acid kit (No. p0009, Beyotime Biotechnology Co., Ltd., Shanghai, China) was utilized to determine the protein concentration. Electrophoresis was then performed in polyacrylamide gel (5% concentrate and 12% separation gel). Tris-buffered saline Tween-20 (TBST) containing 5% bovine serum albumin (BSA) was used to seal the membrane for 1 h in a decolorizing shaker at room temperature. The sealing solution was discarded, and the membrane was put into the plastic groove added with 5% BSA to prepare the primary antibody solution of corresponding concentration overnight at 4 °C. The primary antibodies used are shown in Table 2. The membrane was washed with TBST three times, 10 min each time. Then, the membrane was incubated at 4 °C for 4 h with the secondary antibody solution. The membrane was washed with TBST 3 times, 10 min each time. The membrane was immersed in electrochemiluminescence developer (wbkls0100, Merck Millipore, Billerica, MA, USA) for visualization. The relative optical density (OD) of all immunoblotting bands was analyzed. Western blots were quantified with Image J software (NIH, Bethesda, MD) and normalized to the respective loading controls.

**Table 2:** Antibody sources and dilution rate

Protein name	Company	Cat. No.	Working dilution
RUNX2	Abcam	ab76956	1:1000
Osterix	Abcam	ab22552	1:3000
Osteocalcin	Abcam	ab13418	1:2000
Wnt5a	Proteintech	55184	1:5000
β-catenin	Proteintech	17565	1:2000
p-β-catenin	Abcam	ab73153	1:3000
GAPDH	Proteintech	60004	1:5000

### Dual-luciferase reporter assay

3′-UTR of the GRM5 or LINC00314 gene containing putative miR-129-5p targeting site was amplified by chemical synthesis and was inserted into the psiCHECK2 vector (Promega, Madison, WI, USA). When the confluence was up to 70%, ADSCs were transfected with related mixtures including 50 ng GRM5/LINC00314 wild-type or GRM5/LINC00314 mutant-type 3′-UTR reporter plasmids, miR-129-5p mimics, or miR-129-5p NC in a final concentration of 20 nM, and Lipofectamine 2000 for 48 h. Luciferase activity was detected using the dual-luciferase reporter gene kit (Beyotime, Shanghai, China).

### ALP activity and staining

ADSCs were washed with precooled PBS three times and lysed in precooled 1% Triton X-100 on ice for 30 min. Cell lysate was subjected to ALP activity determination, and the value at 405 nm was normalized to that of total protein concentration.

ADSCs were washed with PBS three times and fixed with 4% paraformaldehyde for 10 min and then added NBT-BCIP solution (BiYunTian Biotech Company, Shanghai, China) for incubation for another 15 min. Images were captured under a microscope (Olympus DP73 Microscope, Olympus, Tokyo, Japan).

### Alizarin red staining (ARS)

ADSCs were induced to undergo osteogenesis for 14 days. Cells were washed, fixed in 95% ethanol for 14 min and dyed with 2% ARS-Tris-HCL solution (pH 4.3). Visible mineralized nodules were captured under an inverted microscope.

### Statistical analysis

All data were processed by SPSS 22.0 statistical software (IBM Corp., Armonk, NY). Data are displayed as the mean ± SD. Comparisons among multiple groups were assessed by one-way analysis of variance. The data at different time points were analyzed by repeated-measures analysis of variance. *P* < 0.05 indicated statistical significance.

## Results

### Identification of ADSCs

**Fig. 1:**
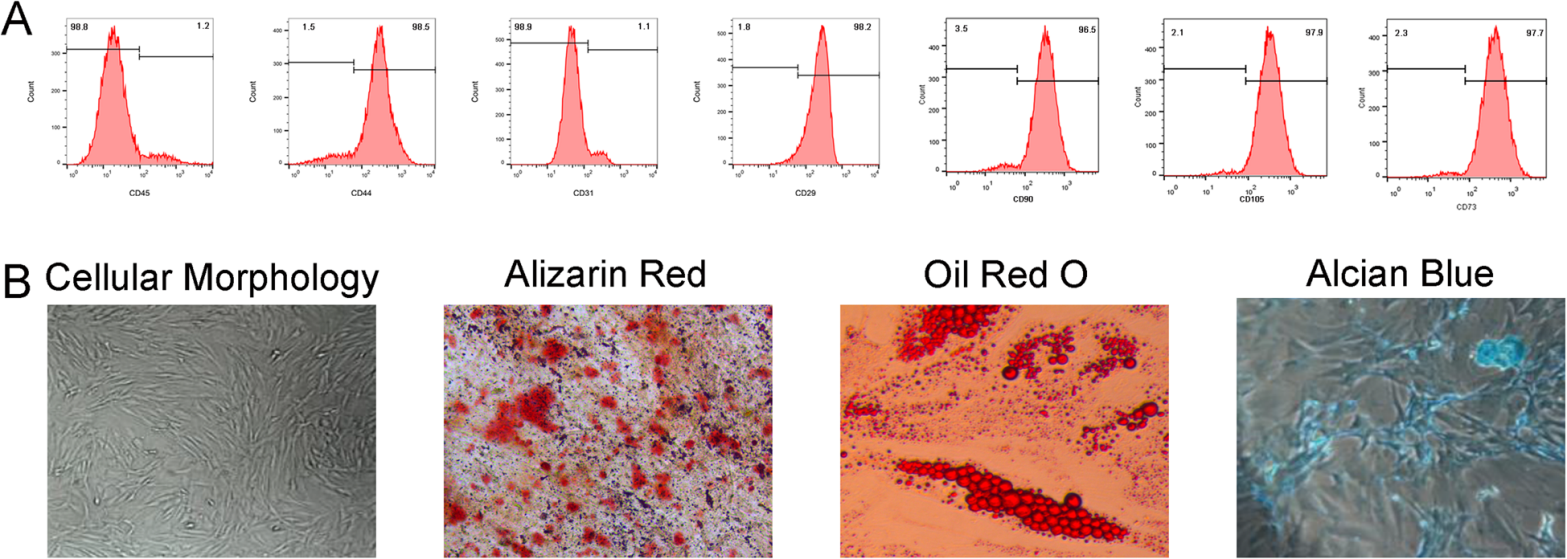
Phenotype identification and trilineage differentiation potential of ADSCs. **a** ADSCs were negative for CD45 and CD31 and positive for CD44 and CD29. **b** ADSCs were induced to undergo osteogenic differentiation, adipogenic differentiation, and chondrogenic differentiation

To identify the phenotype of ADSCs, flow cytometry was used to detect the cell CD markers (CD44, CD29, CD31, CD45, CD73, CD90, and CD105) and trilineage differentiation potential. Flow cytometry results revealed that the ADSCs at passage 3 were negative for CD45 (1.2%) and CD31 (1.1%) and positive for CD44 (98.5%), CD29 (98.2%), CD73 (97.7%), CD 90 (98.5%), and CD 105 (97.8%, Fig. 1a). In addition, ADSCs could successfully differentiate into osteoblasts (ARS, Fig. 1b), adipocytes (Oil Red O, Fig. 1b), and chondrocytes (Alcian blue, Fig. 1b).

### LncRNA LINC00314 is upregulated during ADSCs osteogenic differentiation

**Fig. 2:**
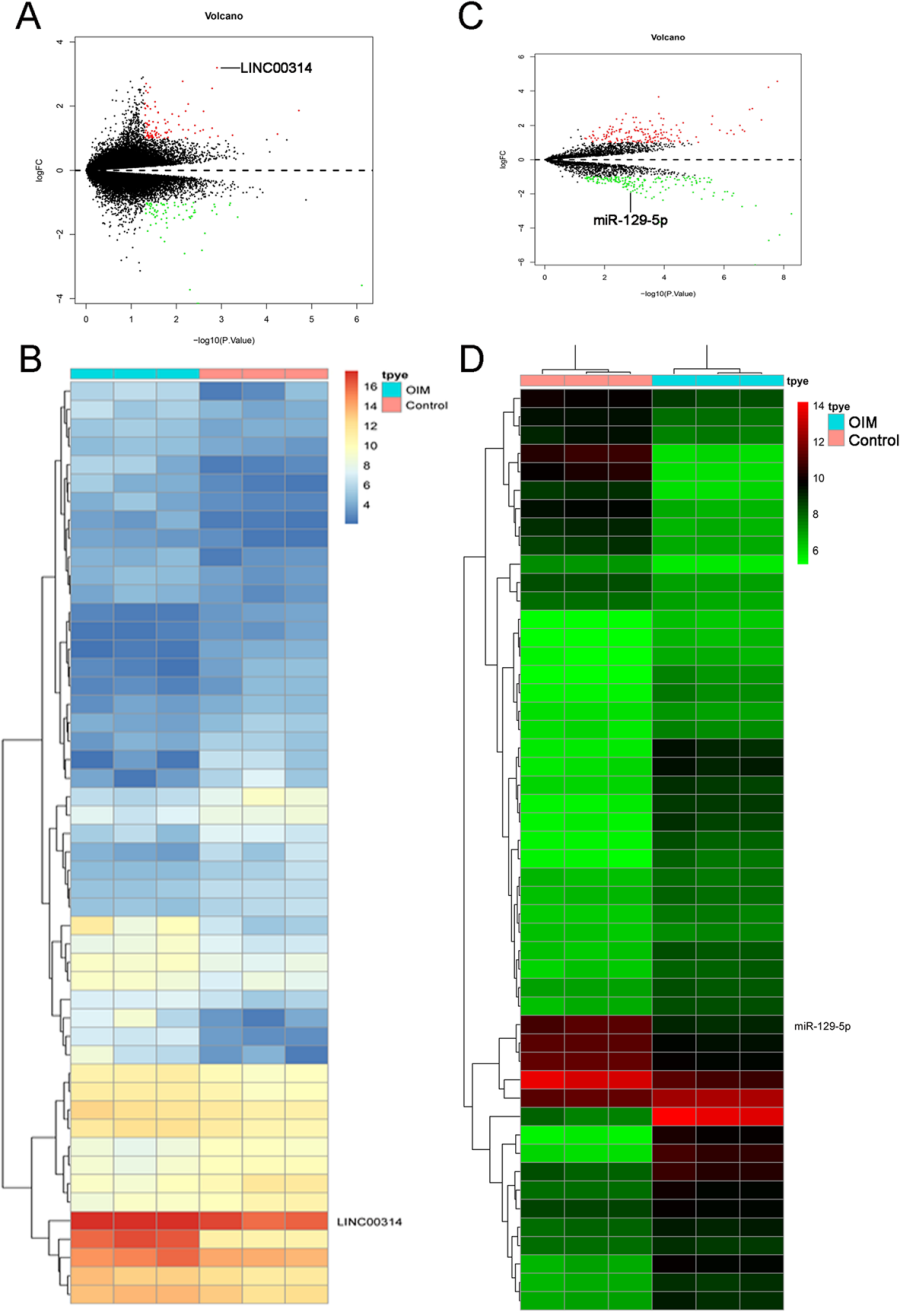
The volcano plots of the significantly differentially expressed (*P* < 0.05, fold change > 2) lncRNAs (**a**). Heatmap of the significantly differentially expressed lncRNAs (**b**). The volcano plots of the significantly differentially expressed (*P* < 0.05, fold change > 2) microRNAs (**c**). Heatmap of the significantly differentially expressed microRNAs (**d**)

To further determine which lncRNA and microRNA participant into the process of ADSCs osteogenic differentiation, differentially altered lncRNA and microRNA in non-induced and induced ADSCs were analyzed further (Fig. 2a). A total of 185 upregulated lncRNAs and 153 downregulated lncRNAs were associated with osteogenic differentiation of ADSCs (*P* < 0.05; logFC> 1, Fig. 2b). Top 100 differentially expressed lncRNAs are presented in Supplementary Table S1. In particular, LINC00314 was found significantly upregulated (logFC = 3.19, *P* = 0.001).

In the microRNA array, a total of 105 microRNAs were identified as differentially expressed. Among these differentially expressed microRNAs, 50 were downregulated and 55 were upregulated (Fig. 2c, d). The top 100 differentially expressed miRNAs between OIM and control groups were listed in Supplementary Table S2. In particular, miR-129-5p was found significantly downregulated (logFC = − 1.93, *P* < 0.05).

### Bioinformatic analysis

**Fig. 3:**
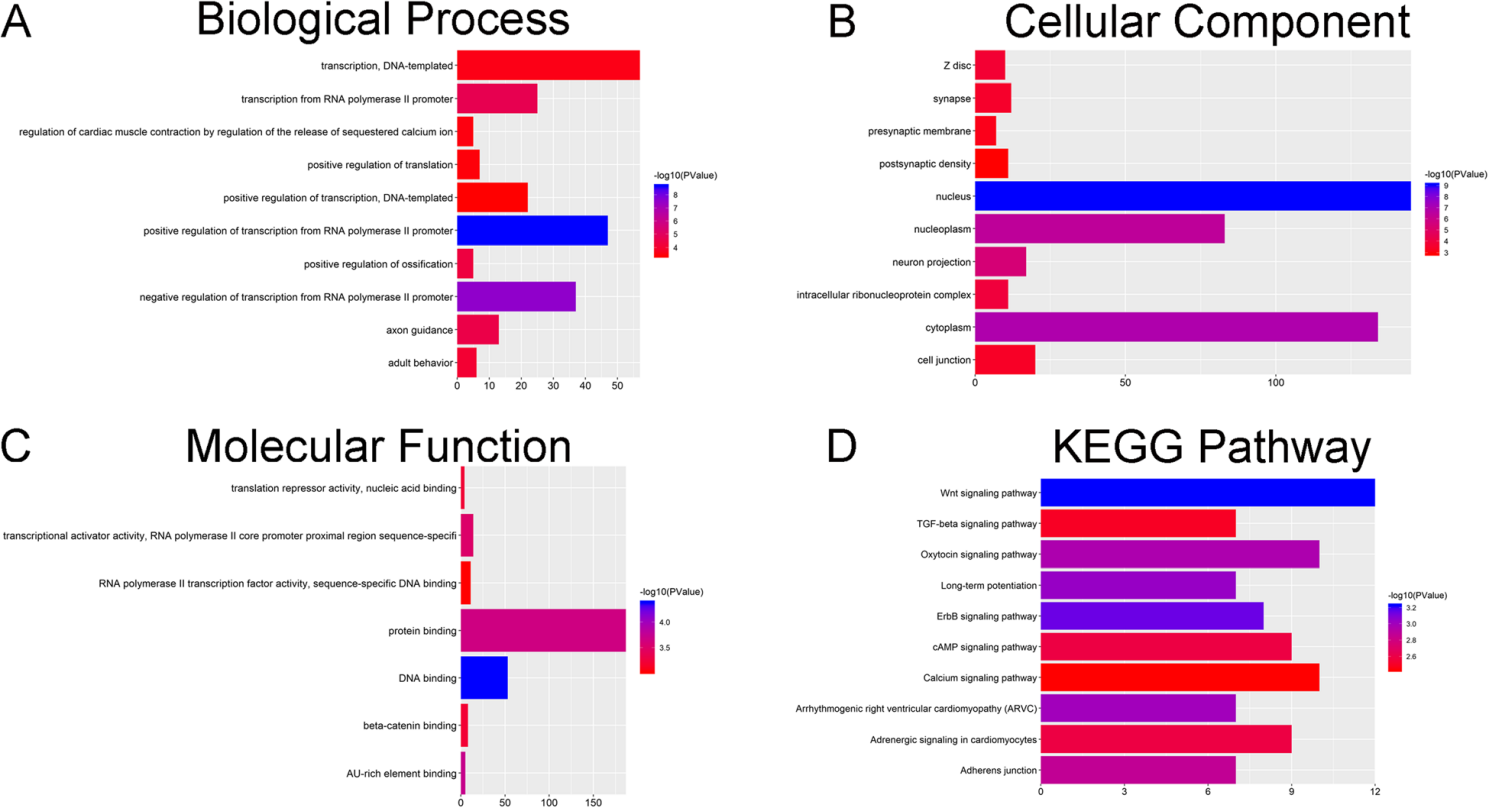
**a** Biological process terms of the target genes of miR-129-5p. **b** Cellular component terms of the target genes of miR-129-5p. **c** Molecular function terms of the target genes of miR-129-5p. **d** KEGG pathway of the target genes of miR-129-5p

Analysis in DAVID was performed using the data profile of differentially expressed mRNAs identified by R software. GO terms that included BP, CC, and MF are listed in Fig. 3a, b, and c, respectively. The most significantly enriched biological processes, cellular component, and molecular function terms were transcription, DNA-templated, Z disc and translation repressor activity, and nucleic acid binding, respectively. In the KEGG pathway analysis, the Wnt/β-catenin signaling pathway was considered to be significantly enriched during osteogenic differentiation of ADSCs (Fig. 3d).

**Fig. 4:**
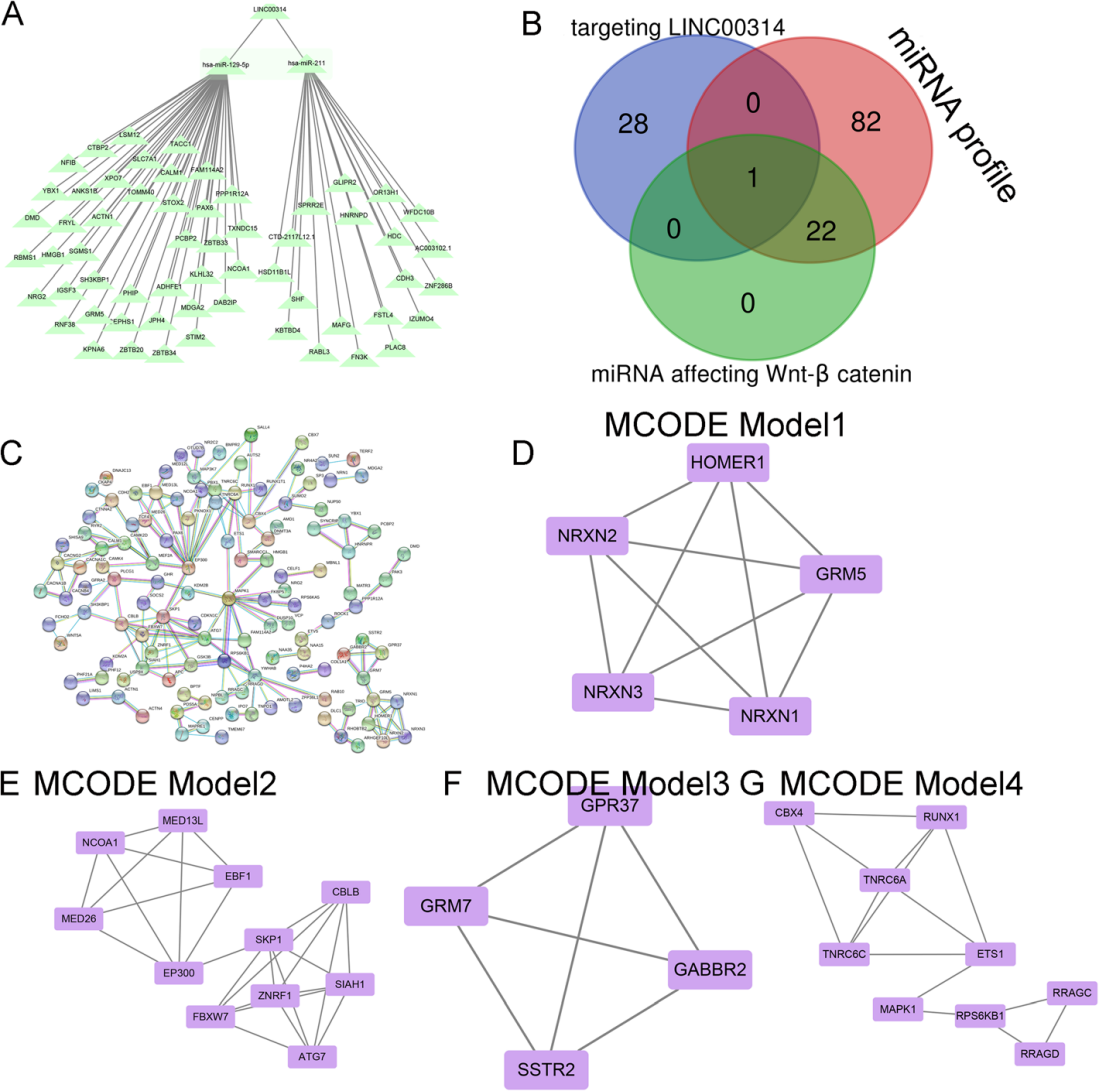
**a** CeRNA network centered on LINC00314, miR-129-5p, and its potential target genes. **b** Venn diagram of miRNAs binding to LINC00314/Wnt/β-catenin and miRNAs from the miRNA profile identified miR-129-5p. **c** Protein-protein interaction of the target genes of miR-129-5p. **d** MCODE model 1, **e** MCODE model 2, **f** MCODE model 3, and **g** MCODE model 4

We then constructed a LINC00314-miRNA-target gene network using Cytoscape to visualize their interrelationships based on our miRNA-seq and target gene data (Fig. 4a). A Venn diagram was generated to assess the overlap between miRNAs targeting LINC00314, the miRNA profile, and miRNAs affecting Wnt/β-catenin signaling, and miR-129-5p was identified (Fig. 4b). A PPI network was constructed to reveal the target gene interaction (Fig. 4c).

The network was then analyzed using the MCODE plugin, and four clustering modules were identified according to the chosen screening conditions. Clustering module 1 scored 5.2 with 5 nodes and 9 edges (Fig. 4d), clustering module 2 scored 4.8 with 11 nodes and 24 edges (Fig. 4e), clustering module 3 scored 4.2 with 4 nodes and 6 edges (Fig. 4f), and clustering module 4 scored 3.5 with 9 nodes and 15 edges (Fig. 4g).

### Effects of LINC00314 on osteogenic differentiation of ADSCs

**Fig. 5:**
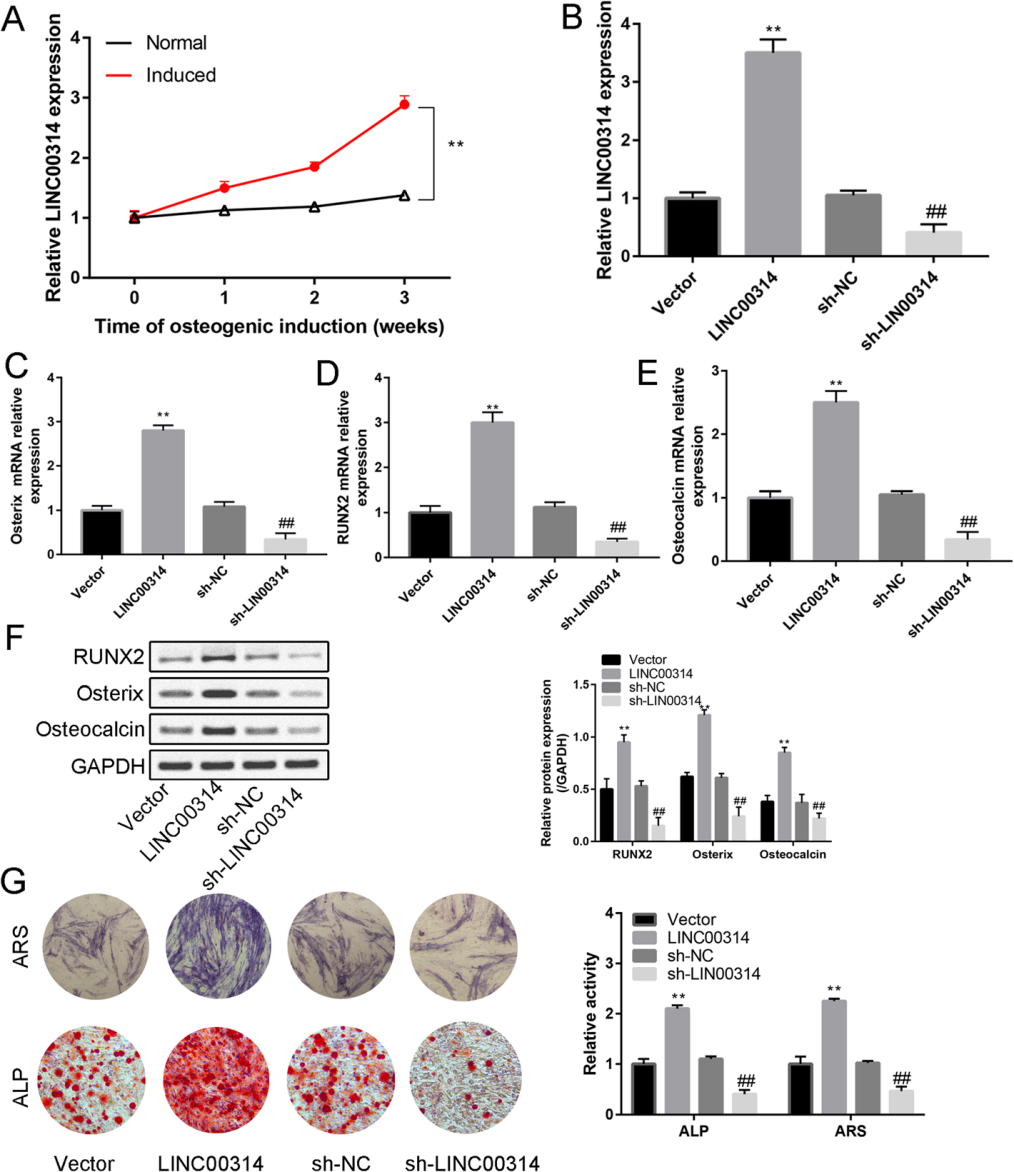
**a** Relative expression of LINC00314 in normal and induced ADSCs from week 1 to week 3. **b** Relative expression of LINC00314 in induced ASCs after pcDNA3.1-LINC00314 or sh-LINC00314 transfection. Relative expression of Runx2 (**c**), Osterix (**d**), and osteocalcin (**e**) after transfection. **f** Western blot analysis of Runx2, Osterix, and osteocalcin after transfection. **g** Alizarin red staining (21 days) and ALP staining (14 days): changes in calcium nodule formation and ALP activity after transfection. **P < 0.01, compared with the NC group; ^##^*P* < 0.01, compared with the sh-NC group

Compared with the normal group, the induced group had significantly increased LINC00314 expression at 1, 2, and 3 weeks (Fig. 5a, *P* < 0.01). The expression of LINC00314 was significantly increased or decreased after transfection of LINC00314 or sh-LINC00314, respectively, and thus the successful plasmid construction was verified (Fig. 5b). Moreover, osteogenic markers (Osterix, RUNX2, and osteocalcin) were significantly upregulated or downregulated after transfection of LINC00314 or sh-LINC00314, respectively (Fig. 5c–f). A large number of calcified nodules were identified by ARS and ALP activity was increased by LINC00314 overexpression, whereas these effects were suppressed by LINC00314 silencing (*P* < 0.05, *P* < 0.01, Fig. 5g). These data indicated that overexpression LINC00314 promoted the osteogenic differentiation of ADSCs.

### LINC00314 act as a sponge of miR-129-5p to stimulate ADSCs osteogenic differentiation

**Fig. 6:**
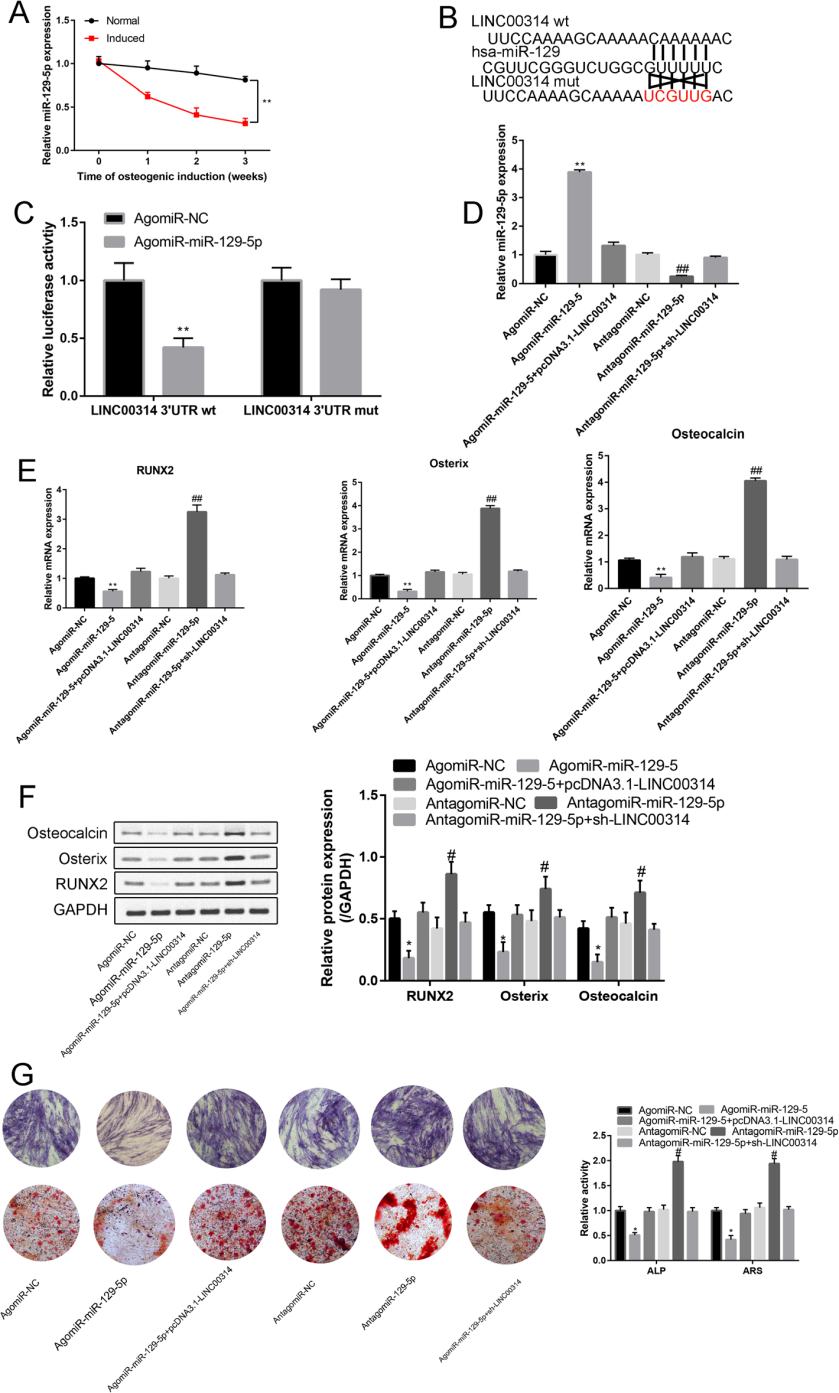
**a** Expression levels of miR-129-5p during the osteoblast differentiation process of ADSCs, as detected by RT-PCR. **b** Binding site between miR-129-5p and the wt/mut LINC00314 3′UTR. **c** Luciferase activity (LINC00314 3′UTR wt and LINC00314 3′UTR mut) in agomiR-NC- and agomiR-miR-129-5p-transfected cells. **d** Relative expression of miR-129-5p in the agomiR-NC, agomiR-miR-129-5p, agomiR-miR-129-5p+pcDNA3.1-LINC00314, antagomiR-NC, antagomiR-miR-129-5p, and antagomiR-miR-129-5p+sh-LINC00314 groups. **e** Relative expression of Runx2, Osterix, and osteocalcin after transfection. **f** Western blot analysis of Runx2, Osterix, and osteocalcin after transfection. **g** ARS and ALP staining in the transfection groups. **P < 0.01, compared with the NC group; ^##^*P* < 0.01, compared with the sh-NC group

The expression level of miR-129-5p exhibited a time-dependent decrease during the osteogenic differentiation process (*P* < 0.01, Fig. 6a). As shown in Supplementary Fig. 1, a significant negative correlation between LINC00314 and miR-129-5 was detected in induced ADSCs at 3 weeks, thus suggesting that LINC00314 may act as a sponge of miR-129-5p. Overexpression of miR-138 attenuated 3′UTR- LINC00314-WT-induced luciferase activity without affecting 3′UTR- LINC00314-MUT-induced luciferase activity (Fig. 6b, c).

Compared with the NC group, agomiR-129-5p significantly increased miR-129-5p expression, while antagomiR-129-5p had the opposite effect (*P* < 0.01, Fig. 6d). These experiments demonstrated the successful transfection efficiency of agomiR-129-5p and antagomiR-129-5p. The effects of increased miR-129-5p expression could be neutralized by pcDNA3.1-LINC00314. The effects of inhibited miR-129-5p expression were neutralized by sh-LINC00314.

The relative expression of Runx2, Osterix, and osteocalcin in the agomiR-129-5p group was significantly downregulated compared with that in the NC group (*P* < 0.01, Fig. 6e, f). Additionally, antagomiR-129-5p significantly increased Runx2, Osterix, and osteocalcin expression compared with the NC group (*P* < 0.01, Fig. 6e, f). This effect was partially reversible by sh-LINC00314.

ARS and ALP staining indicated that agomiR-129-5p significantly decreased calcium nodule formation and ALP activity, and LINC00314 overexpression could neutralize the agomiR-129-5p effects on osteogenic differentiation (*P* < 0.01, Fig. 6g).

### MiR-129-5p regulated ASC osteogenic differentiation through GRM5

**Fig. 7:**
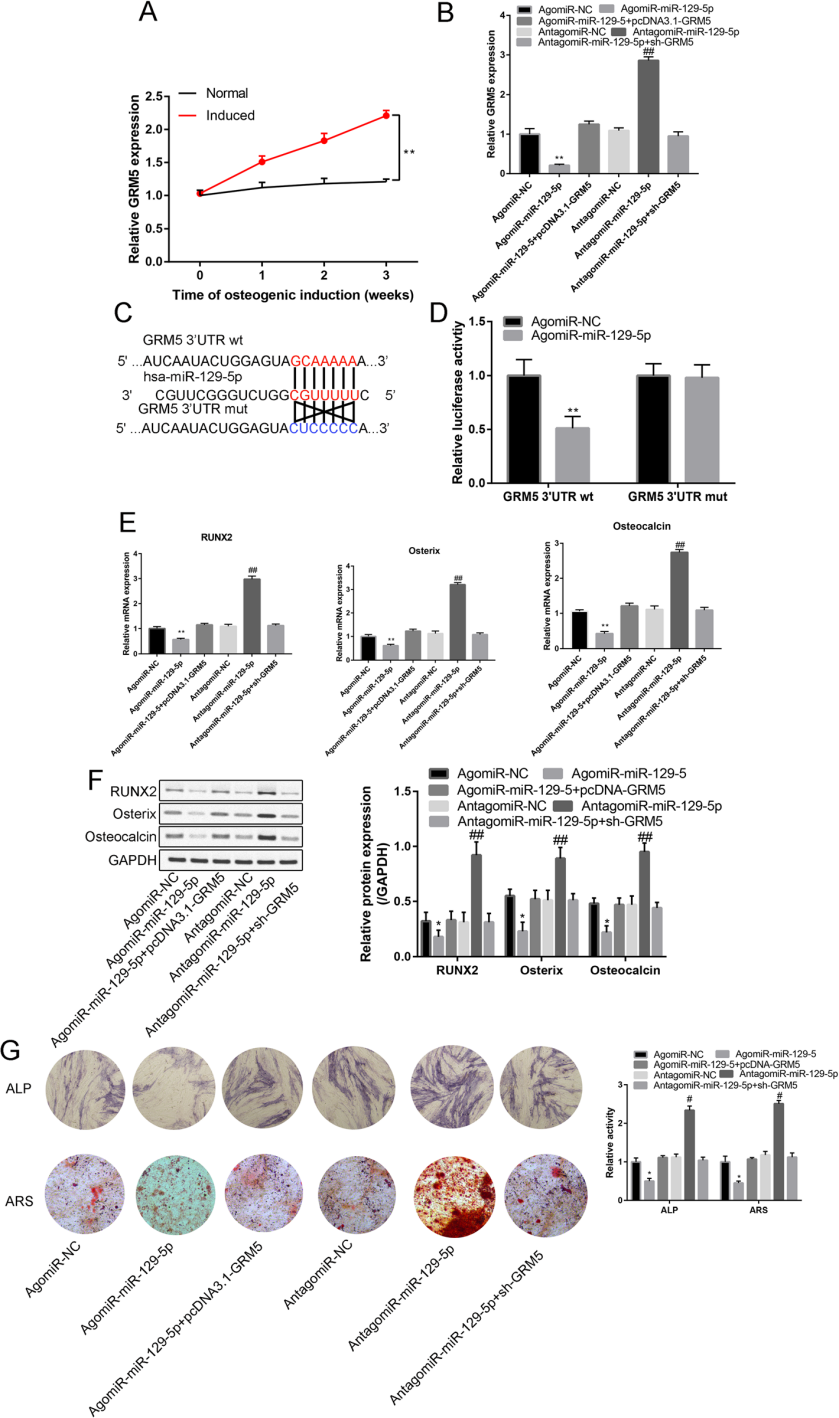
**a** Relative expression of GRM5 in the normal and induced groups from week 1 to week 3. **b** Relative expression of GRM5 in the agomiR-NC, agomiR-miR-129-5p, agomiR-129-5p+pcDNA3.1-GEM5, antagomiR-NC, antagomiR-miR-129-5p, and antagomiR-miR-129-5p+sh-GRM5 groups. **c** Binding site of miR-129-5p and the wt/mut GRM5 3′UTR. **d** Luciferase activity (GRM5 3′UTR wt and GRM5 3′UTR mut) in agomiR-NC- and agomiR-miR-129-5p-transfected cells. **e** Relative expression of Runx2, Osterix, and osteocalcin in the agomiR-NC, agomiR-miR-129-5p, agomiR-129-5p+pcDNA3.1-GEM5, antagomiR-NC, antagomiR-miR-129-5p, and antagomiR-miR-129-5p+sh-GRM5 groups. **f** Western blot analysis of Runx2, Osterix, and osteocalcin in the agomiR-NC, agomiR-miR-129-5p, agomiR-129-5p+pcDNA3.1-GEM5, antagomiR-NC, antagomiR-miR-129-5p, and antagomiR-miR-129-5p+sh-GRM5 groups. **g** ARS and ALP staining in the transfection groups. **P < 0.01, compared with the NC group; ^##^*P* < 0. 01, compared with the sh-NC group

PCR analysis showed that as the induction time increased, the relative GRM5 expression gradually increased (*P* < 0.05) in the induced group compared with the normal group (Fig. 7a). There was a clear significant negative correlation between the miR-129-5p and the GRM5 expression (*R*^2^ = 0.871, *P* = 0.001, Supplementary Fig. 2).

Moreover, compared with the corresponding NC group, agomiR-129-5p significantly inhibited GRM5 expression, while antagomiR-129-5p significantly increased GRM5 expression (Fig. 7b, *P* < 0.05).

Figure 7c presents the binding sites of miR-129-5p on GRM5 3′UTR wt. Furthermore, agomiR-129-5p significantly reduced the relative luciferase activity of the GRM5 3′UTR wt construct (Fig. 7d).

AgomiR-129-5p significantly reduced Runx2, Osterix, and osteocalcin relative expression compared with agomiR-NC (Fig. 7e, f), while the agomiR-129-5p effects on RUNX2, OOX, and OCN relative expression could be reversed by pcDNA-GRM5 and antagomiR-129-5p (Fig. 7e, f).

Figure 7g shows the ARS and ALP results. Compared with the corresponding NC group, miR-129-5p significantly reduced ALP activity and calcium nodule formation. However, these inhibitory effects could be neutralized by GRM5 or antagomir-miR-129-5p (Fig. 7g)

### GRM5 regulated the Wnt/β-catenin signaling pathway

**Fig. 8:**
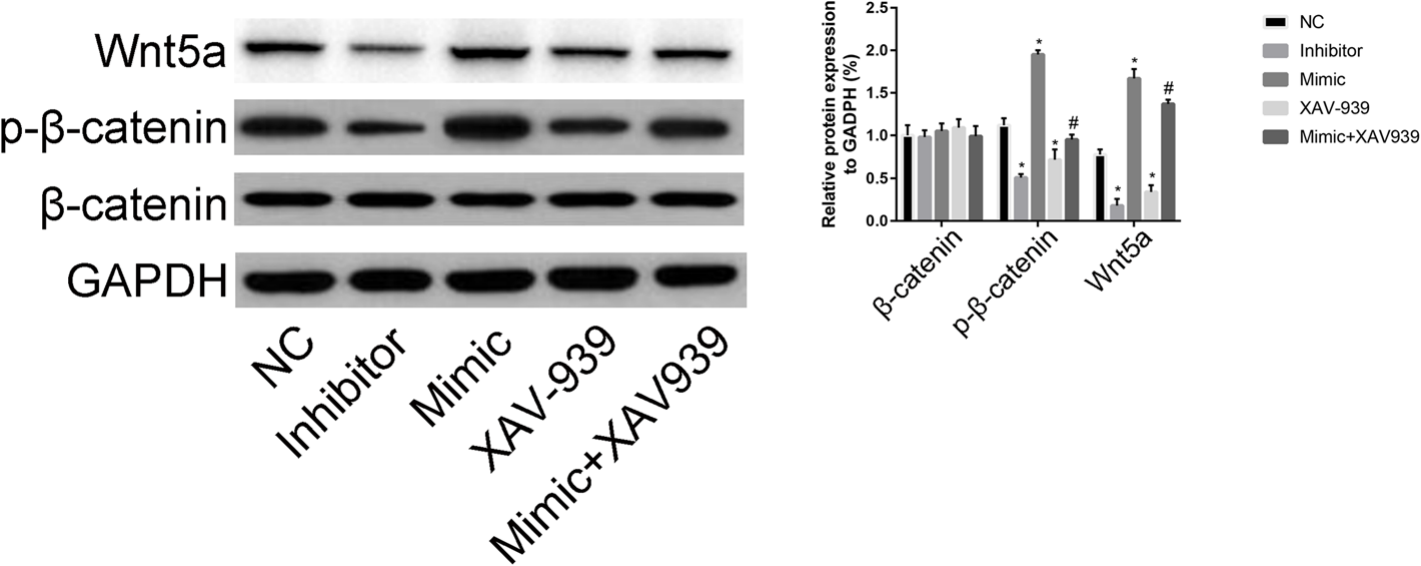
Effects of the miR-129-5p/GRM5 axis on the Wnt/β-catenin signaling pathway. Effects of GRM5 mimic, GRM5 inhibitor, XAV-939, and GRM5 mimic+XAV-939 on the expression of β-catenin, p-β-catenin, and Wnt5a. **P < 0.01, compared with the NC group; ^##^*P* < 0.01, compared with the sh-NC group

The Wnt/β-catenin inhibitor XAV939 (10 μM) was administered to identify the mechanism of action of GRM5 on the Wnt/β-catenin signaling pathway. As illustrated in Fig. 8, we found that, compared with NC, the GRM5 inhibitor was associated with a reduction in p-β-catenin and Wnt5a expression, while GRM5 mimic had the opposite effects on p-β-catenin and Wnt5a expression.

Compared with the mimic group, the GRM5 mimic+XAV939 (10 μM) group was associated with a reduction in p-β-catenin and Wnt5a expression. These findings suggested that GRM5 regulated the Wnt/β-catenin signaling pathway and thus promoted osteogenic differentiation of ADSCs.

## Discussion

We firstly identified LINC00314 plays a critical role in the regulation of osteogenic differentiation of ADSCs. Gain and loss of LINC00314/miR-129-5p/GRM5 function studies revealed that LINC00314 could positively regulate the early and late osteogenesis of ADSCs. Furthermore, the LINC00314/miR-129-5p/GRM5 axis exerts its osteogenic effects on ADSCs through the Wnt/β-catenin signaling pathway.

Recently, ADSCs have gained considerable attention in tissue-engineered bone due to the fat tissue is a rich source of MSCs with minimal injury and less painful [20]. Previous studies have demonstrated that ADSCs can trigger differentiation into adipocytes, chondrocytes, and osteoblasts under specific optimal medium condition [21–23]. Therefore, the promotion of osteogenic differentiation of ADSCs played an important role.

In this study, microarray was firstly conducted to reveal the differentially expressed lncRNAs and identified LINC00314 as the most statistically significant lncRNA. Yi et al. [24] revealed that lncRNA MALAT1 promoted osteogenic differentiation of ADSCs by directly targeting with miR-30. Wu et al. [10] also found that lncRNA HIF1A-AS2 acts as a sponge of miR-665 to regulate IL-6 expression and facilitates bone formation through regulating PI3K/Akt signaling pathway.

We predicted the LINC00314 binding site by using the bioinformatics databases (miRcode). The overlap between the predicted microRNAs and microRNA array results found that miR-129-5p was linked with both LINC00314 and GRM5. Previous studies showed that miR-129-5p function as oncogenes or tumor suppressors during the occurrence and development of multiple cancers, including retinoblastoma [13], non-small cell lung cancer [25], and hepatocellular carcinoma [26]. Its functional role in osteogenic differentiation of ADSCs is poorly demonstrated.

We found that overexpressing miR-129-5p could significantly downregulate ALP activity and calcium deposits. Thus, we concluded that LINC00314 is a sponge of miR-129-5p. Han et al. [27] found that miR-129-5p directly targeting ROCK1 and suppresses osteosarcoma progression, which is consistent with our research findings. Luciferase assays showed that miR-129-5p directly bind to the 3′UTR of GRM5. Finally, GRM5 could regulate the Wnt/β-catenin signaling pathway to induce the osteogenic differentiation of ADSCs. Wnt/β-catenin is a very complex signaling pathway and is involved in ADSC differentiation [28]. Xu et al. [29] found miR-889 directly targeting WNT7A and subsequently suppressing bone formation.

The Wnt/β-catenin signaling pathway is closely related to osteoblast differentiation of ADSCs. Anabolic agents that stimulate the Wnt/β-catenin signaling pathway can be used to treat osteoblast-related diseases such as osteoporosis. Recently, antisclerostin and anti-dickkopf antibodies were used to conduct the preliminary clinical trial and show great potential for the treatment of osteoporosis [30–32]. Western blotting and loss/gain-of-function assays of the Wnt pathway confirmed that upregulated expression of GRM5 induced the activity of the Wnt signaling pathway, which promoted the osteogenic differentiation of ADSCs.

## Conclusions

**Fig. 9:**
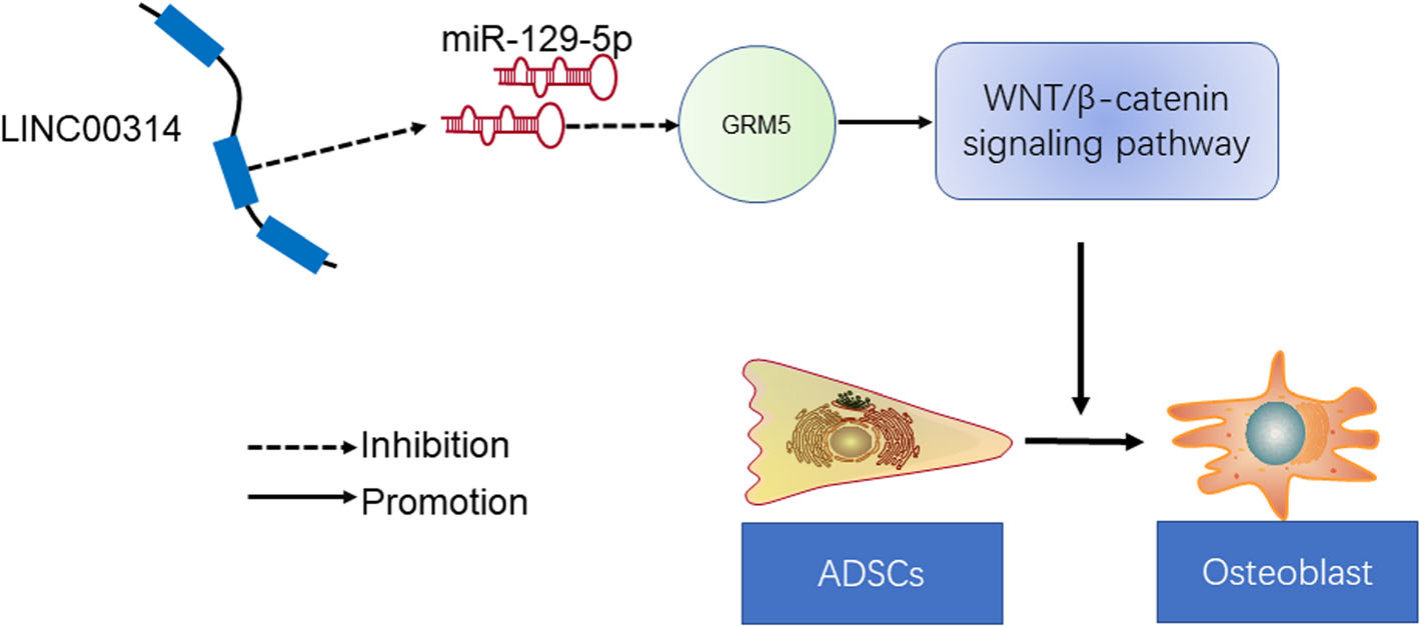
Schematic diagram of lncRNA LINC00314/miR-129-5p/GRM5 in osteogenic differentiation of ADSCs

In conclusion, we propose that the LINC00314/miR-129-5p/GRM5 axis positively regulates the early and late osteogenic differentiation ability by modulating the Wnt/β-catenin signaling pathway in ADSCs (Fig. 9). LINC00314 was upregulated during osteogenic differentiation of ADSCs, which enhanced its miR-129-5p sponge function, thereby promoting GRM5 expression by activating the Wnt/β-catenin signaling pathway. Our findings could provide a new target for controlling osteogenesis in ADSCs, which are crucial to bone tissue engineering and treatment for bone diseases.

## Supplementary information

**Additional file 1: Supplement Figure 1.** Relationship between LINC00314 and miR-129-5p in ADSCs after osteogenic differentiation for 3 weeks.

**Additional file 2: Supplement Figure 2.** Relationship between miR-129-5p and GRM5 in ADSCs after osteogenic differentiation for 3 weeks.

**Additional file 3: Supplementary Table S1.** Differentially expressed lncRNAs between induced and non-induced ADSCs. logFC: log fold change; AveExpr: average expression; adj. P.Val: adjustive *P* value.

**Additional file 4: Supplementary Table S2.** Differentially expressed miRNAs between induced and non-induced ADSCs. logFC: log fold change; AveExpr: average expression; adj. P.Val: adjustive *P* value.

## Data Availability

The datasets used and analyzed during the current study are available from the corresponding author on reasonable request.

## Abbreviations

ADSCs: Adipose-derived stem cells
lncRNAs: Long noncoding RNAs
ARS: Alizarin red staining
ALP: Alkaline phosphatase
BMD: Bone mineral density
BMSCs: Bone marrow mesenchymal stem cells
miRNA: microRNA
FBS: Fetal bovine serum
DMEM: Dulbecco’s modified Eagle medium
OIM: Osteogenic induction medium
BSA: Bovine serum albumin
DAVID: Database for Annotation, Visualization and Integrated Discovery
GO: Gene ontology
KEGG: Kyoto Encyclopedia of Genes and Genomes
BP: Biological process
CC: Cellular component
MF: Molecular function
STRING: Search Tool for the Retrieval of Interacting Genes
PPI: Protein-protein interaction
cDNA: Complementary deoxyribose nucleic acid
PBS: Phosphate-buffered saline
