# Supplementary figures and images for: Long noncoding RNA LINC00314 facilitates osteogenic differentiation of adipose-derived stem cells through the hsa-miR-129-5p/GRM5 axis via the Wnt signaling pathway
Source: Stem Cell Res Ther. 2020 Jun 17;11:240. doi: 10.1186/s13287-020-01754-z (PMC7302136; doi:10.1186/s13287-020-01754-z)

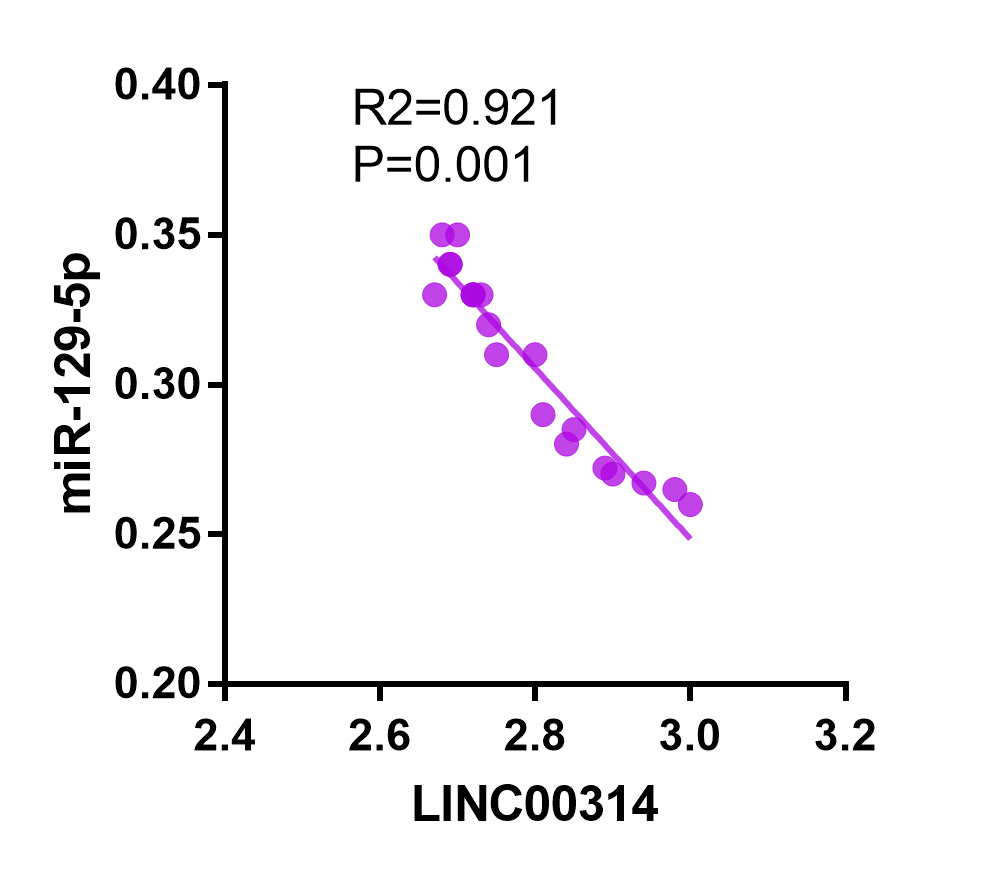

Supplement: Supplementary file 1 — Additional file 1: Supplement Figure 1. Relationship between LINC00314 and miR-129-5p in ADSCs after osteogenic differentiation for 3 weeks. [file 13287_2020_1754_MOESM1_ESM.tif]

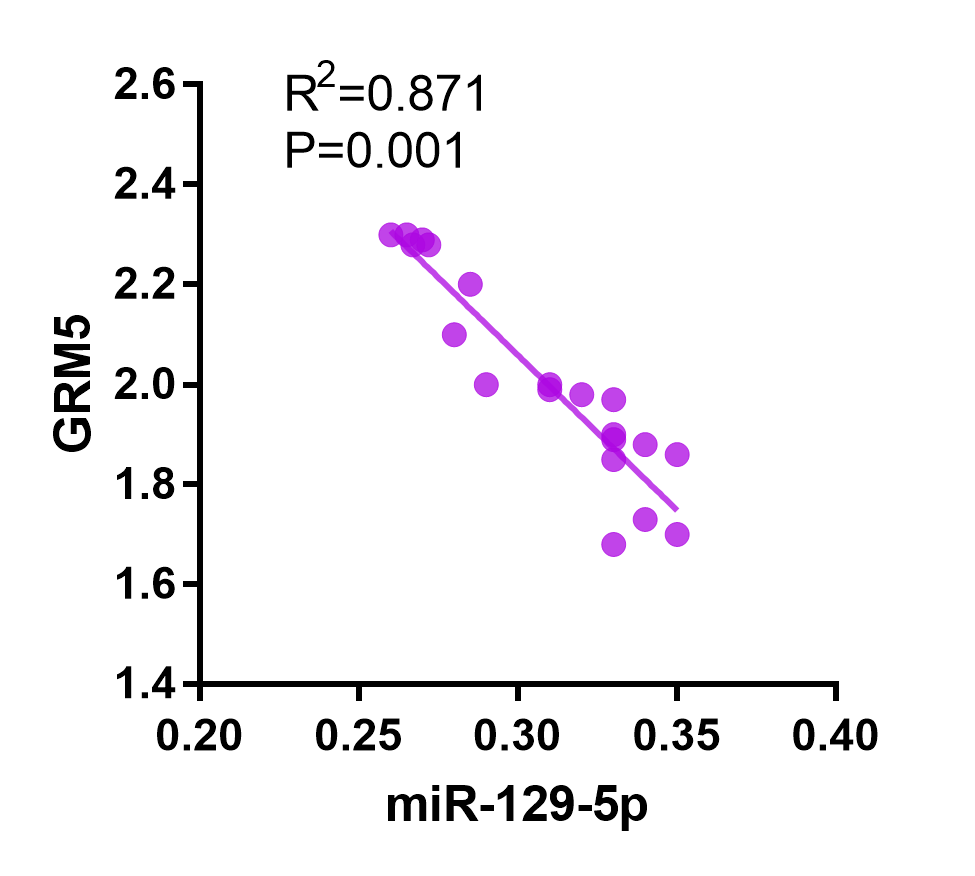

Supplement: Supplementary file 2 — Additional file 2: Supplement Figure 2. Relationship between miR-129-5p and GRM5 in ADSCs after osteogenic differentiation for 3 weeks. [file 13287_2020_1754_MOESM2_ESM.tif]
