# Supplementary material for: Long noncoding RNA LINC00314 facilitates osteogenic differentiation of adipose-derived stem cells through the hsa-miR-129-5p/GRM5 axis via the Wnt signaling pathway
Source: Stem Cell Res Ther. 2020 Jun 17;11:240. doi: 10.1186/s13287-020-01754-z (PMC7302136; doi:10.1186/s13287-020-01754-z)
Supplement: Supplementary file 3 — Additional file 3: Supplementary Table S1. Differentially expressed lncRNAs between induced and non-induced ADSCs. logFC: log fold change; AveExpr: average expression; adj. P.Val: adjustive P value. [file 13287_2020_1754_MOESM3_ESM.docx]

| Gene | logFC | AveExpr | t | P.Value | adj.P.Val | B |
| --- | --- | --- | --- | --- | --- | --- |
| AC004982 | -3.59 | 5.01 | -23.6642 | 7.75E-07 | 0.05463 | -2.08437 |
| AC078828 | 1.857222 | 3.221389 | 13.22619 | 1.92E-05 | 0.451927 | -2.18549 |
| CCDC3 | 1.136111 | 5.660833 | 10.80865 | 5.75E-05 | 0.785311 | -2.25419 |
| MYPN | -1.45611 | 8.6675 | -7.36024 | 0.000438 | 0.785311 | -2.46849 |
| AC011389 | 1.099444 | 3.101389 | 6.997633 | 0.000567 | 0.785311 | -2.50695 |
| RAI1-AS1 | -1.02056 | 4.590833 | -6.83775 | 0.000638 | 0.785311 | -2.52544 |
| AC025154 | -1.08306 | 3.986528 | -6.79009 | 0.000661 | 0.785311 | -2.53115 |
| ZNF385D | 1.072222 | 5.514444 | 6.032911 | 0.001197 | 0.785311 | -2.63579 |
| LINC00314 | 3.192778 | 4.270833 | 5.971674 | 0.001259 | 0.785311 | -2.64555 |
| AL392086 | -1.36556 | 3.131111 | -5.94909 | 0.001283 | 0.785311 | -2.6492 |
| HECW1-IT1 | -1.13556 | 11.47889 | -5.84693 | 0.001397 | 0.785311 | -2.66611 |
| AC116563 | -1.06 | 4.245556 | -5.8068 | 0.001445 | 0.785311 | -2.67292 |
| LINC00707 | -1.25111 | 10.38833 | -5.68511 | 0.001603 | 0.785311 | -2.69422 |
| ATP13A4-AS1 | 2.548333 | 7.303611 | 5.684559 | 0.001604 | 0.785311 | -2.69432 |
| VCAN-AS1 | 1.320833 | 8.959583 | 5.677812 | 0.001613 | 0.785311 | -2.69553 |
| AL049795 | 1.035556 | 2.928333 | 5.546185 | 0.001809 | 0.785311 | -2.71973 |
| LINGO1-AS1 | -1.96389 | 3.569722 | -5.2635 | 0.002327 | 0.785311 | -2.77591 |
| PGM5-AS1 | 1.834444 | 3.480556 | 5.188588 | 0.002492 | 0.785311 | -2.79183 |
| LINC01133 | 1.265833 | 4.525972 | 5.165959 | 0.002545 | 0.785311 | -2.79673 |
| AC007092 | -2.49167 | 3.879167 | -5.09797 | 0.00271 | 0.785311 | -2.8117 |
| AC113146 | 1.233611 | 9.37625 | 5.004581 | 0.002958 | 0.785311 | -2.83291 |
| AC026461 | -4.15611 | 5.293056 | -4.91849 | 0.003209 | 0.785311 | -2.85315 |
| AL356124 | -4.15694 | 7.800694 | -4.84108 | 0.003456 | 0.785311 | -2.87193 |
| LINC00115 | 1.165 | 5.166944 | 4.730967 | 0.003846 | 0.785311 | -2.89963 |
| AL109924 | -1.30889 | 10.66167 | -4.72823 | 0.003856 | 0.785311 | -2.90033 |
| LINC02389 | 1.406667 | 4.658333 | 4.721851 | 0.00388 | 0.785311 | -2.90198 |
| AF015262 | -1.24389 | 12.40417 | -4.7082 | 0.003933 | 0.785311 | -2.90551 |
| NNMT | -1.31556 | 3.727222 | -4.67017 | 0.004083 | 0.785311 | -2.91544 |
| IGF2 | -1.00722 | 3.778056 | -4.65438 | 0.004147 | 0.785311 | -2.9196 |
| NPY2R | -1.13722 | 11.75167 | -4.57107 | 0.004505 | 0.785311 | -2.94201 |
| AC022809 | -1.4325 | 6.486806 | -4.5692 | 0.004513 | 0.785311 | -2.94253 |
| TRA2A | -1.43306 | 14.53097 | -4.53665 | 0.004663 | 0.785311 | -2.95149 |
| SYN2 | -1.49111 | 4.899444 | -4.48952 | 0.00489 | 0.785311 | -2.96466 |
| AC068790 | -3.72583 | 5.195694 | -4.47026 | 0.004986 | 0.785311 | -2.97012 |
| LINC02216 | -1.45333 | 3.591111 | -4.43798 | 0.005153 | 0.785311 | -2.97935 |
| LINC02336 | 1.266667 | 7.714444 | 4.388655 | 0.005419 | 0.785311 | -2.99369 |
| PRXL2A | 2.061389 | 4.881806 | 4.380166 | 0.005467 | 0.785311 | -2.99618 |
| PAPPA2 | -1.24556 | 16.86139 | -4.30031 | 0.005937 | 0.785311 | -3.02006 |
| NR2F1 | -1.05444 | 12.97028 | -4.25706 | 0.006211 | 0.785311 | -3.03331 |
| AC073525 | 1.016667 | 4.02 | 4.256421 | 0.006215 | 0.785311 | -3.03351 |
| AC004585 | 1.841111 | 10.30944 | 4.255526 | 0.006221 | 0.785311 | -3.03379 |
| DEXI | -2.59444 | 4.668889 | -4.20398 | 0.006566 | 0.785311 | -3.04988 |
| GPC6-AS1 | -1.53278 | 7.730278 | -4.13317 | 0.007077 | 0.785311 | -3.07251 |
| KRT7-AS | -1.07111 | 3.107222 | -4.13302 | 0.007079 | 0.785311 | -3.07257 |
| AL050338 | 1.271944 | 9.84375 | 4.12574 | 0.007134 | 0.785311 | -3.07493 |
| AL022724 | 2.77 | 4.676667 | 4.110448 | 0.007251 | 0.785311 | -3.07991 |
| HMOX1 | -1.38722 | 8.409722 | -3.93852 | 0.00873 | 0.785311 | -3.13805 |
| AL162231 | 1.428611 | 3.52125 | 3.932041 | 0.008792 | 0.785311 | -3.14032 |
| AC018904 | 1.688333 | 6.276389 | 3.925757 | 0.008853 | 0.785311 | -3.14252 |
| AC124854 | -1.12889 | 11.40556 | -3.89959 | 0.009111 | 0.785311 | -3.15176 |
| AL138759 | -1.09056 | 4.741944 | -3.74267 | 0.010846 | 0.785311 | -3.20915 |
| ELK3 | -1.00778 | 4.857778 | -3.73081 | 0.010992 | 0.785311 | -3.21363 |
| AC009884 | -1.48444 | 3.260556 | -3.72588 | 0.011053 | 0.785311 | -3.2155 |
| AC008537 | 1.760556 | 4.196944 | 3.628878 | 0.012338 | 0.785311 | -3.25297 |
| LINC00856 | 1.211389 | 12.17042 | 3.583001 | 0.013003 | 0.785311 | -3.27118 |
| DACT1 | 1.265556 | 3.882778 | 3.490405 | 0.014471 | 0.785311 | -3.3089 |
| LINC02407 | 1.033333 | 5.532222 | 3.44195 | 0.015312 | 0.785311 | -3.32917 |
| AP000462 | 1.030278 | 12.27792 | 3.440257 | 0.015342 | 0.785311 | -3.32989 |
| AL049838 | -1.16306 | 6.557083 | -3.4394 | 0.015358 | 0.785311 | -3.33025 |
| PCBP2 | 1.514167 | 12.17431 | 3.432099 | 0.01549 | 0.785311 | -3.33334 |
| EMX2OS | 1.196667 | 4.898889 | 3.408338 | 0.015928 | 0.785311 | -3.34344 |
| LINC02397 | -1.78833 | 4.6275 | -3.40265 | 0.016034 | 0.785311 | -3.34588 |
| BMP6 | -1.03361 | 11.16903 | -3.39816 | 0.016119 | 0.785311 | -3.3478 |
| AC074286 | -1.48778 | 3.780556 | -3.38588 | 0.016354 | 0.785311 | -3.35308 |
| CLVS2 | -1.63722 | 8.8775 | -3.3718 | 0.016628 | 0.785311 | -3.35916 |
| AC011139 | 1.168056 | 9.250972 | 3.371684 | 0.01663 | 0.785311 | -3.35921 |
| SLC7A11 | -1.31194 | 12.01958 | -3.35668 | 0.016928 | 0.785311 | -3.36573 |
| AC139718 | 1.4675 | 10.00403 | 3.336435 | 0.017339 | 0.785311 | -3.37458 |
| AC092834 | -1.24806 | 10.38347 | -3.32366 | 0.017604 | 0.785311 | -3.3802 |
| LINC01611 | 1.185556 | 12.23222 | 3.320968 | 0.01766 | 0.785311 | -3.38138 |
| AL031727 | 1.585833 | 11.56125 | 3.302189 | 0.01806 | 0.785311 | -3.3897 |
| MANCR | 1.003333 | 3.800556 | 3.294917 | 0.018217 | 0.785311 | -3.39293 |
| ART3 | -2.21972 | 7.498194 | -3.29065 | 0.01831 | 0.785311 | -3.39484 |
| AC211486 | -1.07472 | 13.63875 | -3.27953 | 0.018555 | 0.785311 | -3.39981 |
| LINC01013 | -1.05778 | 7.358889 | -3.2536 | 0.01914 | 0.785311 | -3.41147 |
| MGME1 | -1.34889 | 8.719444 | -3.24293 | 0.019386 | 0.785311 | -3.4163 |
| AC106744 | -1.07139 | 10.28458 | -3.20831 | 0.020211 | 0.785311 | -3.43211 |
| AC092902 | -1.52056 | 6.399167 | -3.16096 | 0.021403 | 0.785311 | -3.45406 |
| EIF4E2 | -1.02444 | 12.35833 | -3.15111 | 0.021661 | 0.785311 | -3.45867 |
| NEDD9 | -1.03 | 9.128889 | -3.15082 | 0.021668 | 0.785311 | -3.4588 |
| LINC01270 | -1.03 | 9.128889 | -3.15082 | 0.021668 | 0.785311 | -3.4588 |
| KANSL1-AS1 | -2.09694 | 10.73319 | -3.13627 | 0.022055 | 0.785311 | -3.46564 |
| ZRANB2 | 1.092222 | 11.55556 | 3.136266 | 0.022055 | 0.785311 | -3.46564 |
| AC009505 | 1.040833 | 4.642083 | 3.064117 | 0.024089 | 0.785311 | -3.50008 |
| AC003973 | 1.135556 | 6.555556 | 3.045854 | 0.024636 | 0.785311 | -3.50893 |
| FOXD1-AS1 | 1.803056 | 13.15958 | 3.02334 | 0.02533 | 0.785311 | -3.51992 |
| USP2-AS1 | 1.496111 | 8.798611 | 3.020871 | 0.025407 | 0.785311 | -3.52114 |
| AC112722 | -1.26444 | 11.35194 | -3.00395 | 0.025944 | 0.785311 | -3.52946 |
| AC239804 | 1.025556 | 14.25111 | 2.978576 | 0.026773 | 0.785311 | -3.54203 |
| MVB12A | -1.41278 | 15.92111 | -2.97617 | 0.026853 | 0.785311 | -3.54323 |
| CCDC80 | 1.185 | 7.989167 | 2.969067 | 0.027091 | 0.785311 | -3.54677 |
| LINC02537 | -1.08333 | 5.708333 | -2.96035 | 0.027386 | 0.785311 | -3.55113 |
| AL139317 | -1.08333 | 5.708333 | -2.96035 | 0.027386 | 0.785311 | -3.55113 |
| AC100800 | -1.30556 | 5.635556 | -2.95764 | 0.027478 | 0.785311 | -3.55249 |
| AC012613 | 1.17 | 8.227778 | 2.945932 | 0.027882 | 0.785311 | -3.55836 |
| AL357514 | 1.313056 | 8.897083 | 2.941435 | 0.028038 | 0.785311 | -3.56063 |
| CREB5 | 1.344722 | 4.37125 | 2.938534 | 0.02814 | 0.785311 | -3.56209 |
| IGF2-AS | 1.111389 | 8.220417 | 2.93579 | 0.028236 | 0.785311 | -3.56347 |
| TMEM71 | -1.65722 | 8.280278 | -2.93548 | 0.028247 | 0.785311 | -3.56363 |
| AC048341 | 1.088889 | 7.861111 | 2.924156 | 0.028649 | 0.785311 | -3.56936 |

Supplementary Table S1: Differentially expressed lncRNAs between induced and non-induced ADSCs. logFC: log fold change; AveExpr: average expression; adj.P.Val: adjustive P value.
