# Supplementary material for: Long noncoding RNA LINC00314 facilitates osteogenic differentiation of adipose-derived stem cells through the hsa-miR-129-5p/GRM5 axis via the Wnt signaling pathway
Source: Stem Cell Res Ther. 2020 Jun 17;11:240. doi: 10.1186/s13287-020-01754-z (PMC7302136; doi:10.1186/s13287-020-01754-z)
Supplement: Supplementary file 4 — Additional file 4: Supplementary Table S2. Differentially expressed miRNAs between induced and non-induced ADSCs. logFC: log fold change; AveExpr: average expression; adj. P.Val: adjustive P value. [file 13287_2020_1754_MOESM4_ESM.docx]

| Gene | logFC | AveExpr | t | P.Value | adj.P.Val | B |
| --- | --- | --- | --- | --- | --- | --- |
| hsa-miR-4478 | -3.1698 | 7.133718 | -47.1858 | 5.57E-09 | 1.91E-05 | 11.26831 |
| hsa-let-7a-3p | -4.40612 | 7.757098 | -40.5019 | 1.40E-08 | 1.91E-05 | 10.58302 |
| hsa-miR-301b-3p | 4.558235 | 8.227024 | 39.37893 | 1.65E-08 | 1.91E-05 | 10.44894 |
| hsa-miR-500a-5p | -4.73103 | 8.30892 | -35.2988 | 3.19E-08 | 2.28E-05 | 9.905539 |
| hsa-miR-629-3p | 4.212374 | 7.994381 | 35.13549 | 3.28E-08 | 2.28E-05 | 9.88176 |
| hsa-miR-4651 | 2.323402 | 7.800111 | 32.12795 | 5.62E-08 | 3.25E-05 | 9.412085 |
| hsa-miR-3151-3p | -2.69917 | 6.815351 | -29.8799 | 8.68E-08 | 3.96E-05 | 9.016997 |
| hsa-miR-664a-5p | -6.16392 | 10.75678 | -29.6321 | 9.13E-08 | 3.96E-05 | 8.970877 |
| hsa-miR-4701-5p | 2.966645 | 7.374674 | 28.5795 | 1.13E-07 | 4.37E-05 | 8.768814 |
| hsa-miR-210-3p | 2.911898 | 8.134357 | 27.59823 | 1.40E-07 | 4.85E-05 | 8.571028 |
| hsa-miR-2392 | -3.46874 | 7.362139 | -26.5783 | 1.75E-07 | 5.44E-05 | 8.355133 |
| hsa-miR-629-5p | 1.661311 | 8.580025 | 25.82463 | 2.08E-07 | 5.44E-05 | 8.188416 |
| hsa-miR-5006-5p | -3.0251 | 8.198298 | -25.6686 | 2.16E-07 | 5.44E-05 | 8.153107 |
| hsa-miR-126-3p | 1.739736 | 10.38591 | 25.15199 | 2.44E-07 | 5.44E-05 | 8.034146 |
| hsa-miR-199b-5p | -3.14143 | 7.109916 | -25.0987 | 2.47E-07 | 5.44E-05 | 8.021689 |
| hsa-miR-3648 | -2.74479 | 7.379653 | -25.0285 | 2.51E-07 | 5.44E-05 | 8.005245 |
| hsa-miR-335-5p | 1.998425 | 7.745023 | 24.3462 | 2.96E-07 | 6.05E-05 | 7.842163 |
| hsa-miR-329-3p | -3.42401 | 7.565412 | -22.9301 | 4.24E-07 | 8.17E-05 | 7.484389 |
| hsa-miR-4656 | 1.490383 | 7.674164 | 22.52527 | 4.71E-07 | 8.46E-05 | 7.376997 |
| hsa-miR-214-5p | -1.96975 | 7.321209 | -22.3956 | 4.88E-07 | 8.46E-05 | 7.342085 |
| hsa-miR-874-3p | -2.11654 | 7.802921 | -22.0056 | 5.42E-07 | 8.95E-05 | 7.235606 |
| hsa-miR-34a-3p | 1.71194 | 10.54211 | 21.67338 | 5.93E-07 | 9.36E-05 | 7.143039 |
| hsa-miR-4532 | -1.87142 | 7.153972 | -20.7093 | 7.78E-07 | 0.000115 | 6.864394 |
| hsa-miR-221-3p | 1.535507 | 6.421006 | 20.62846 | 7.96E-07 | 0.000115 | 6.840333 |
| hsa-miR-196a-5p | -1.87627 | 6.294652 | -20.0516 | 9.43E-07 | 0.000131 | 6.665248 |
| hsa-miR-378a-3p | -2.34815 | 6.607434 | -19.4799 | 1.12E-06 | 0.00015 | 6.485746 |
| hsa-miR-1290 | 1.723656 | 8.420691 | 18.75133 | 1.41E-06 | 0.000181 | 6.247892 |
| hsa-miR-129-5p | -1.93802 | 8.778419 | -17.4293 | 2.17E-06 | 0.000269 | 5.787888 |
| hsa-miR-100-5p | -2.12692 | 7.902278 | -17.252 | 2.30E-06 | 0.000276 | 5.723192 |
| hsa-miR-4725-5p | 2.547376 | 12.18907 | 17.05485 | 2.47E-06 | 0.000285 | 5.650389 |
| hsa-miR-4668-5p | 2.076287 | 10.07975 | 16.89997 | 2.60E-06 | 0.000289 | 5.592528 |
| hsa-miR-487b-3p | -1.18013 | 5.951076 | -16.8315 | 2.67E-06 | 0.000289 | 5.566749 |
| hsa-miR-483-3p | -1.32931 | 6.937357 | -16.5588 | 2.94E-06 | 0.000309 | 5.46297 |
| hsa-miR-369-5p | -1.31753 | 7.540196 | -16.4096 | 3.10E-06 | 0.000317 | 5.405362 |
| hsa-miR-342-3p | -1.06182 | 6.835682 | -16.262 | 3.27E-06 | 0.000324 | 5.347842 |
| hsa-miR-3651 | -1.31281 | 6.020623 | -15.9672 | 3.64E-06 | 0.000351 | 5.231169 |
| hsa-miR-412-5p | -1.72711 | 7.445109 | -15.3434 | 4.61E-06 | 0.000426 | 4.976334 |
| hsa-miR-7641 | -1.08216 | 9.09964 | -15.3114 | 4.67E-06 | 0.000426 | 4.962983 |
| hsa-miR-10a-5p | -2.30431 | 9.282535 | -14.8246 | 5.65E-06 | 0.000503 | 4.755753 |
| hsa-miR-4485-3p | -1.28264 | 7.122768 | -14.4914 | 6.46E-06 | 0.000551 | 4.609673 |
| hsa-miR-29b-1-5p | -1.10809 | 6.483318 | -14.471 | 6.51E-06 | 0.000551 | 4.600612 |
| hsa-miR-197-3p | 1.014474 | 7.314233 | 14.17555 | 7.35E-06 | 0.000608 | 4.467888 |
| hsa-miR-630 | -1.38357 | 7.179708 | -13.8672 | 8.37E-06 | 0.000661 | 4.326209 |
| hsa-miR-8485 | -1.31798 | 8.571503 | -13.8636 | 8.38E-06 | 0.000661 | 4.324529 |
| hsa-miR-210-5p | 1.32726 | 9.178778 | 13.79092 | 8.64E-06 | 0.000661 | 4.290659 |
| hsa-miR-505-5p | -1.22678 | 11.96779 | -13.7608 | 8.75E-06 | 0.000661 | 4.276575 |
| hsa-miR-22-5p | -1.08312 | 5.812943 | -13.5335 | 9.65E-06 | 0.000699 | 4.169158 |
| hsa-miR-6716-3p | -1.91223 | 6.442792 | -13.2477 | 1.09E-05 | 0.000775 | 4.031356 |
| hsa-miR-136-3p | -1.02107 | 8.290928 | -13.1943 | 1.12E-05 | 0.000778 | 4.005303 |
| hsa-miR-1207-5p | -1.48246 | 6.591491 | -13.0621 | 1.19E-05 | 0.000804 | 3.940268 |
| hsa-miR-572 | -1.31123 | 6.370974 | -13.0318 | 1.20E-05 | 0.000804 | 3.925266 |
| hsa-miR-24-1-5p | -1.11667 | 6.300358 | -12.708 | 1.40E-05 | 0.000897 | 3.762708 |
| hsa-miR-148b-3p | -1.00076 | 6.009177 | -12.4374 | 1.58E-05 | 0.000963 | 3.623552 |
| hsa-miR-1225-5p | 1.11047 | 13.81074 | 12.29529 | 1.69E-05 | 0.001012 | 3.549251 |
| hsa-miR-26b-5p | -1.06871 | 6.495129 | -12.2133 | 1.76E-05 | 0.001035 | 3.505979 |
| hsa-miR-503-5p | -1.21164 | 5.740256 | -12.0221 | 1.93E-05 | 0.00111 | 3.403939 |
| hsa-miR-324-5p | 1.164173 | 8.471353 | 11.9983 | 1.95E-05 | 0.00111 | 3.391139 |
| hsa-miR-6087 | -1.10063 | 7.113774 | -11.8641 | 2.08E-05 | 0.001122 | 3.318426 |
| hsa-miR-192-5p | 1.317968 | 7.628527 | 11.83289 | 2.12E-05 | 0.001122 | 3.301367 |
| hsa-miR-29c-3p | 1.425149 | 12.54828 | 11.75779 | 2.19E-05 | 0.001122 | 3.260194 |
| hsa-miR-10b-5p | 1.163546 | 6.342436 | 11.75316 | 2.20E-05 | 0.001122 | 3.257648 |
| hsa-miR-374b-5p | 1.080361 | 6.31498 | 11.70854 | 2.25E-05 | 0.001122 | 3.233056 |
| hsa-miR-654-5p | -1.02019 | 6.908861 | -11.6954 | 2.26E-05 | 0.001122 | 3.225768 |
| hsa-miR-100-3p | -1.51364 | 6.095799 | -11.639 | 2.33E-05 | 0.001127 | 3.19455 |
| hsa-miR-324-3p | 2.154195 | 13.21282 | 11.61958 | 2.35E-05 | 0.001127 | 3.183738 |
| hsa-miR-17-3p | 1.171762 | 14.02765 | 11.51816 | 2.47E-05 | 0.001145 | 3.127058 |
| hsa-miR-450a-5p | 1.335165 | 12.64054 | 11.38097 | 2.65E-05 | 0.001195 | 3.0496 |
| hsa-miR-5703 | 1.602417 | 9.869557 | 11.23234 | 2.86E-05 | 0.001273 | 2.964636 |
| hsa-miR-424-5p | 1.011302 | 6.008387 | 11.15137 | 2.98E-05 | 0.001311 | 2.917885 |
| hsa-miR-6724-5p | 1.076329 | 12.055 | 11.08374 | 3.09E-05 | 0.001341 | 2.878579 |
| hsa-miR-148a-3p | 1.731165 | 10.0766 | 11.04887 | 3.15E-05 | 0.001349 | 2.858227 |
| hsa-miR-137 | -1.17679 | 7.583005 | -10.999 | 3.23E-05 | 0.001368 | 2.829012 |
| hsa-miR-432-5p | 1.262825 | 13.98297 | 10.95915 | 3.30E-05 | 0.00138 | 2.805559 |
| hsa-miR-665 | -1.18674 | 7.208759 | -10.7526 | 3.68E-05 | 0.001487 | 2.682681 |
| hsa-miR-19b-1-5p | 1.399248 | 12.39752 | 10.72804 | 3.73E-05 | 0.001489 | 2.667941 |
| hsa-miR-23a-5p | 2.020528 | 8.999628 | 10.63256 | 3.93E-05 | 0.001545 | 2.61025 |
| hsa-miR-32-5p | -1.44727 | 7.113474 | -10.5234 | 4.17E-05 | 0.001608 | 2.543692 |
| hsa-miR-493-3p | -1.11368 | 8.508751 | -10.3785 | 4.52E-05 | 0.001715 | 2.454288 |
| hsa-miR-889-3p | 1.089466 | 10.7723 | 10.33296 | 4.63E-05 | 0.001715 | 2.425939 |
| hsa-miR-933 | 1.455272 | 11.9167 | 9.911521 | 5.88E-05 | 0.001954 | 2.15786 |
| hsa-miR-214-3p | 1.761535 | 12.80896 | 9.894094 | 5.94E-05 | 0.001954 | 2.146544 |
| hsa-miR-222-5p | 1.078279 | 6.37904 | 9.889165 | 5.96E-05 | 0.001954 | 2.14334 |
| hsa-miR-193a-5p | -1.01889 | 7.527105 | -9.78922 | 6.32E-05 | 0.00203 | 2.078048 |
| hsa-miR-25-3p | 1.164633 | 8.792639 | 9.613523 | 7.01E-05 | 0.002191 | 1.961748 |
| hsa-miR-146b-5p | 1.097353 | 13.76682 | 9.58375 | 7.13E-05 | 0.002199 | 1.941844 |
| hsa-miR-4672 | -1.65051 | 7.084197 | -9.57497 | 7.17E-05 | 0.002199 | 1.935964 |
| hsa-miR-410-3p | 1.213248 | 13.84804 | 9.418475 | 7.87E-05 | 0.002336 | 1.830299 |
| hsa-miR-193a-3p | -1.02456 | 6.302688 | -9.37113 | 8.10E-05 | 0.002344 | 1.79801 |
| hsa-miR-339-3p | 1.068129 | 8.419653 | 9.303301 | 8.45E-05 | 0.002419 | 1.751492 |
| hsa-miR-197-5p | 1.597849 | 8.987051 | 9.292288 | 8.50E-05 | 0.002419 | 1.743911 |
| hsa-miR-1185-5p | -1.18699 | 8.958774 | -9.21188 | 8.93E-05 | 0.002501 | 1.688299 |
| hsa-miR-186-5p | -1.76361 | 10.04751 | -9.18523 | 9.08E-05 | 0.002503 | 1.669771 |
| hsa-miR-195-5p | 1.098482 | 6.366975 | 9.176948 | 9.13E-05 | 0.002503 | 1.664 |
| hsa-miR-4484 | 1.289393 | 7.413063 | 9.124825 | 9.43E-05 | 0.002509 | 1.627586 |
| hsa-miR-3940-5p | 1.273546 | 11.56135 | 9.105428 | 9.54E-05 | 0.002509 | 1.613986 |
| hsa-miR-485-3p | 1.030857 | 13.90615 | 8.998269 | 0.000102 | 0.002592 | 1.538376 |
| hsa-miR-628-5p | 1.10366 | 14.36312 | 8.986245 | 0.000103 | 0.002592 | 1.529841 |
| hsa-miR-505-3p | 1.603446 | 11.43457 | 8.961574 | 0.000104 | 0.002592 | 1.512296 |
| hsa-miR-199a-5p | 2.394589 | 13.50273 | 8.932622 | 0.000106 | 0.002619 | 1.491651 |
| hsa-miR-1246 | 1.243384 | 12.13586 | 8.874676 | 0.00011 | 0.002673 | 1.450149 |

Supplementary Table S2: Differentially expressed miRNAs between induced and non-induced ADSCs. logFC: log fold change; AveExpr: average expression; adj.P.Val: adjustive P value.
